# Surface-based molecular self-assembly: Langmuir-Blodgett films of amphiphilic Ln(III) complexes

**DOI:** 10.1186/s13065-016-0224-6

**Published:** 2016-11-28

**Authors:** Dominic J. Wales, Jonathan A. Kitchen

**Affiliations:** Chemistry, University of Southampton, Southampton, Hampshire SO17 1BJ UK

**Keywords:** Lanthanides, Langmuir, Langmuir-Blodgett, Surface, Sensors, Self-assembly, Amphiphilic, Luminescence, Ln(III)

## Abstract

The unique photophysical properties of the Ln(III) series has led to significant research efforts being directed towards their application in sensors. However, for “real-life” applications, these sensors should ideally be immobilised onto surfaces without loss of function. The Langmuir-Blodgett (LB) technique offers a promising method in which to achieve such immobilisation. This mini-review focuses on synthetic strategies for film formation, the effect that film formation has on the physical properties of the Ln(III) amphiphile, and concludes with examples of Ln(III) LB films being used as sensors.

## Background

The construction of lanthanide-based functional nanostructures is an active area of research. Trivalent lanthanide ions have readily manipulated coordination environments and interesting photophysical properties (e.g. sharp, long-lived emission at long wavelengths) making them particularly useful in molecular recognition and sensing [1–5]. The majority of studies have been carried out in solution, however to progress towards practical, robust and commercialised sensing applications (e.g. personal sensors or medical devices) these complexes should ideally be on a surface. As such there has been significant effort directed towards functionalising Ln(III) complexes with groups for surface attachment, including the formation of amphiphilic Ln(III) systems for Langmuir-Blodgett (LB) deposition.

**Fig. 1:**
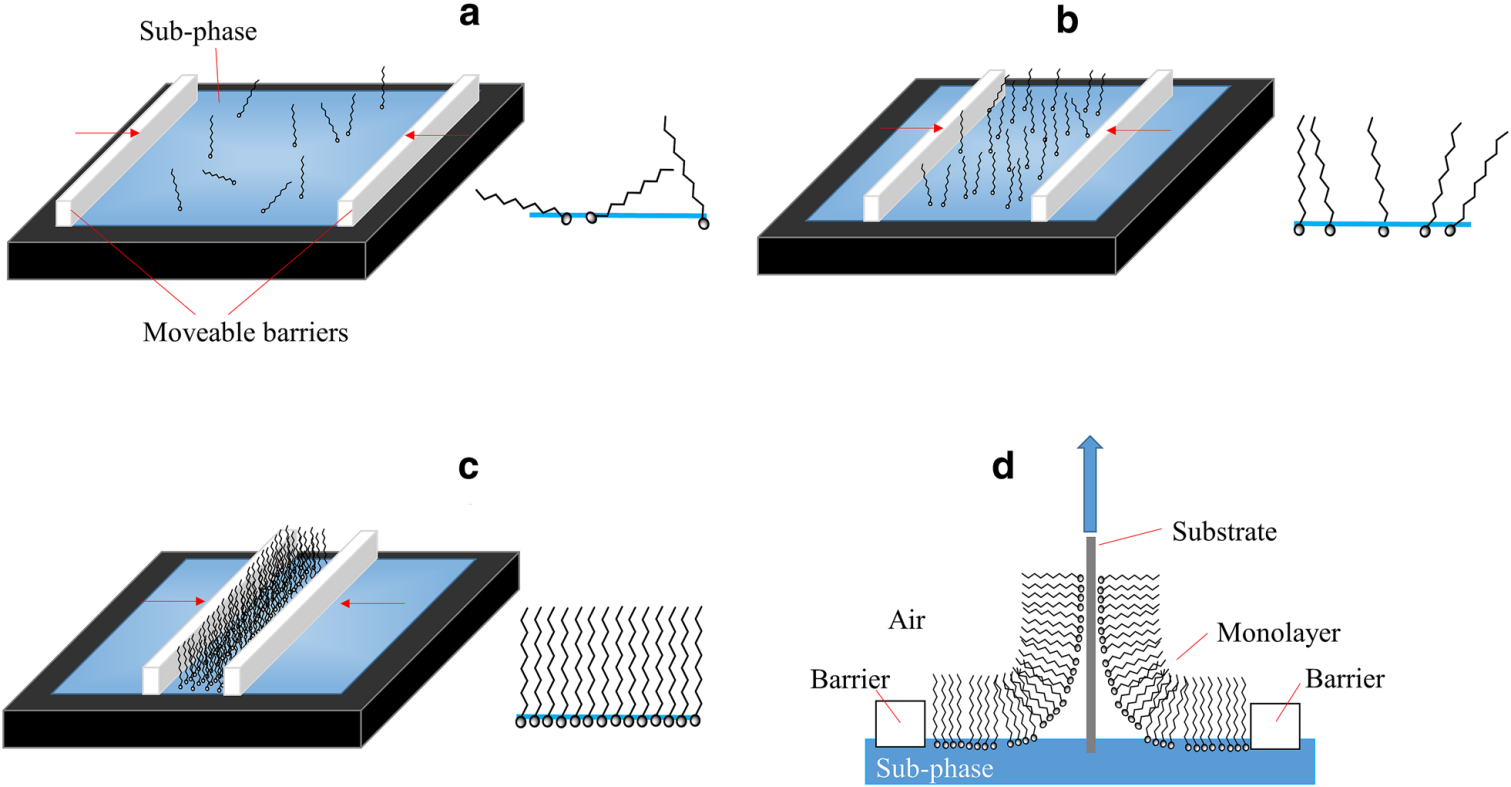
Schematic showing the steps involved in formation of Langmuir-Blodgett films. Each image shows the trough set-up and a side-on view of the interface. **a** Amphiphile is spread onto the sub-phase on a Langmuir trough resulting in a 2D ‘gaseous’ arrangement of amphiphiles (i.e. no interactions between molecules). **b** Barriers are compressed to reduce the surface area of the interface and molecules begin to interact forming a 2D ‘liquid expanded’ phase. **c** On further compression the amphiphiles are self-assembled into a monolayer forming a 2D ‘liquid compressed’ phase. **d** When a monolayer has formed it can be transferred onto a solid support via vertical deposition. *Red arrows* indicate barrier movement direction

The Langmuir-Blodgett technique [6] involves the self-assembly of *amphiphilic* molecules into an ordered mono-layer (Langmuir film) at an interface (usually air/water) and subsequent transfer (via vertical deposition) of the self-assembled mono-layer onto a solid substrate (Langmuir-Blodgett film)—see Fig. 1. The LB technique is an excellent method for depositing self-assembled systems onto surfaces. It offers homogeneity over relatively large areas, and unlike traditional self-assembled monolayers (SAMs), films of multiple layers (including those where each layer has a different composition) can be achieved by successive dipping. When coupled with the unique photophysical properties of the Ln(III) ions the LB technique allows for the development of new generation sensors that allow for sensing on surface rather than the traditional solution based approach, thus allowing the development of functional sensing devices.

## Synthesis of Ln(III) amphiphiles and strategies in film formation

**Fig. 2:**
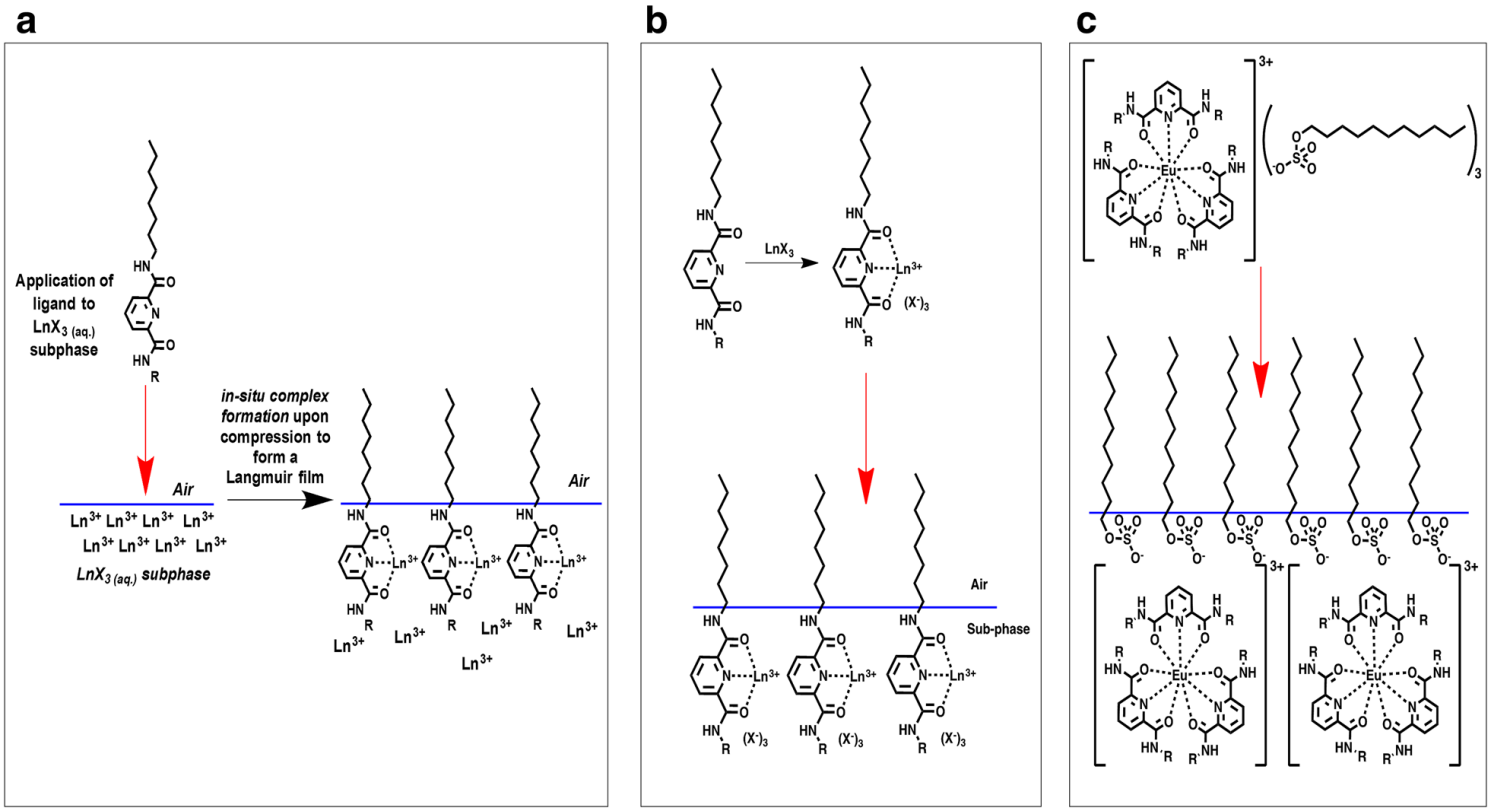
Schematic showing the three methods to prepare Ln(III) amphiphiles. **a** In situ formation—a free ligand is applied to the surface of a Ln(III) containing sub-phase. As the barriers are compressed the ligands coordinate to the Ln(III) in the sub-phase and form a complex. **b** Pre-formed complexes—an amphiphilic ligand is first complexed with Ln(III) and then the resulting amphiphilic complex is applied to the surface of the LB trough. **c** Ln(III) complexes with amphiphilic counter ions—in these systems the counter ion (anion or cation) has amphiphilic character and the ion-pair formed is applied to the surface of the LB trough

Three main methods have been employed to generate Langmuir (and subsequently Langmuir-Blodgett) films from amphiphilic Ln(III) compounds (Fig. 2). For example *pre*-*formed* amphiphilic Ln(III) complexes can be deposited onto a sub-phase (usually pure water) before transfer to a solid support or conversely, the complex can be formed in situ.

In this case the sub-phase of the LB trough contains Ln(III) ions and the amphiphilic free ligands are deposited on the sub-phase to complex with the Ln(III) ions at the air water interface. The last example (which will not be discussed in this review due to space limitations) involves ion-pair systems where ionic Ln(III) complexes contain *amphiphilic counter*-*ions* (e.g. anionic or cationic surfactants outside of the Ln(III) coordination sphere) [7, 8]. Again, due to the need for brevity, this review does not discuss the work on Langmuir-Blodgett films of Ln(III) bisphthalocyanines complexes, as this body of work has been thoroughly reviewed by Rodríguez-Mendez in 2009 and, to the best of our knowledge, there have been no reports of such systems since then [9].

**Fig. 3:**
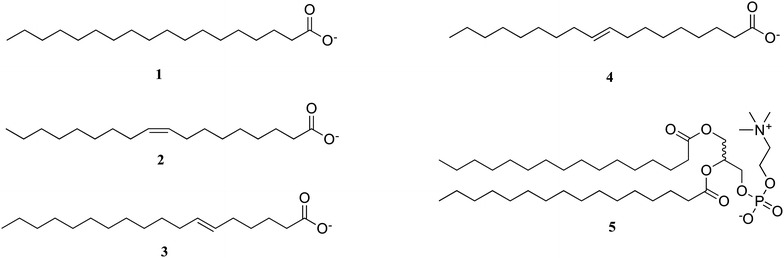
Ligands **1**–**5** used for the in situ formation of Ln(III) LB films

**Fig. 4:**
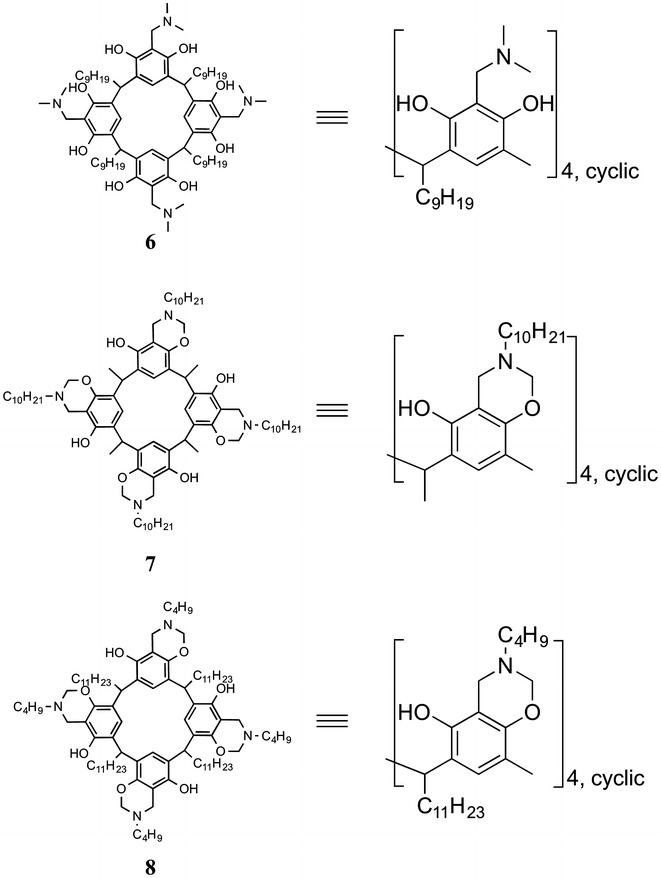
Calix[4]resorcinarene derivatives **6**–**8** investigated by Neveshkin et al

Many of the initial studies in this field focused solely on the film forming abilities of Ln(III) systems utilising the in situ approach. In these studies, fatty acids and fatty acid phosphate esters (Fig. 3) were deposited onto aqueous sub-phases containing Ln(III) cations. These ‘preliminary’ studies have been pivotal to the further development of more advanced Ln(III) based functional materials, despite these initial systems not being luminescent. They have given information pertaining to design requirements for developing ligands (e.g. chain length), deposition conditions (e.g. expected isotherms) and characterisation methods for LB films. Some notable examples of in situ film formation include those of Linden and Rosenholm who prepared Tb(III) containing Langmuir films of simple long chain acids **1**–**4** [10] and Chunbo and co-workers who characterised striped domain Eu(III) containing LB films of **5** on mica using AFM [11]. The previous ligands were not ideal for Ln(III) sensitisation, therefore Neveshkin and co-workers replaced the acid groups with larger, more complex chromophore containing calix[4]resorcinarene derivatives **6**–**8** (Fig. 4) to form Langmuir films on Ln(III) containing sub-phases [12].

## Effect of film formation on Ln(III) emission

With sensing applications in mind, it is important to determine what effects (if any) the arrangement of Ln(III) ions in an ordered LB film has on the physical properties (i.e. emission properties) of the complex. The LB technique results in high local concentrations of amphiphiles in close proximity to a surface, therefore for Ln(III) containing films the biggest concern, especially if they are to be used as a sensor, is quenching of emission. A small number of studies have been carried out that investigated how film formation effected emission properties of the Ln(III) ions within the film.

**Fig. 5:**
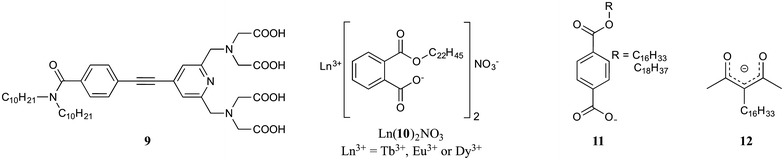
Ligands **9**–**12**

Lemmetyinen and co-workers conducted time-resolved studies into the mechanism of the energy transfer from ligand **9** (Fig. 5) to Eu(III) or Tb(III) ions in LB films [13]. The energy transfer between **9** and Eu(III) and Tb(III) took place in the solid LB films with high efficiency, and following direct comparisons between energy transfer in solution and in the film, they concluded that in both cases energy transfer occurred via similar mechanisms. Xu and co-workers prepared amphiphilic complexes of Tb(III), Dy(III) and Eu(III) using **10** (Fig. 5) [14]. Solutions of the three pre-formed lanthanide complexes, [Ln(**10**)_2_NO_3_], were deposited onto pure water sub-phases and LB films prepared. Efficient emission from LB films of [Tb(**10**)_2_NO_3_] and [Dy(**10**)_2_NO_3_] were observed with characteristics similar to the bulk solids. However, in LB films of [Eu(**10**)_2_NO_3_] the emission was much weaker, likely ascribed to the triplet state energy of **10** being less efficient at sensitising Eu(III) compared to Tb(III) and Dy(III). The same group also reported the in situ fabrication and subsequent emission properties of LB films of Eu(III) and Dy(III) complexes of **11** (Fig. 5) [15]. Serra and co-workers investigated the in situ formation of Eu(III) complexes of the amphiphilic β-diketonate ligand **12** (Fig. 5) [16]. The multi-layered (3 layers) LB film obtained displayed the characteristic emission associated with Eu(III) and was similar to solution and solid-state emission measurements of [Eu(**12**)_6_].

**Fig. 6:**
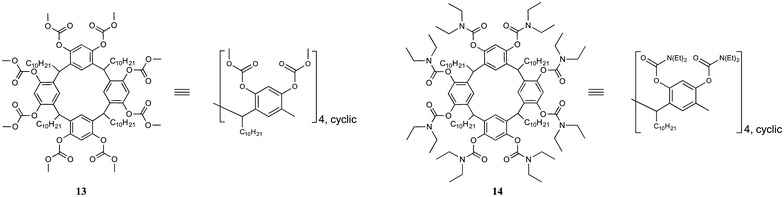
Calix[4]resorcinarene derivatives **13** and **14** investigated by Zaniquelli et al

Whilst the above results suggest that LB film formation has little to no effect on the quantum yield or emission properties of the Ln(III) systems, Zaniquelli showed otherwise with investigations using in situ formed of multi-layered Tb(III) films of **13** and **14** (Fig. 6) [17]. LB films of these systems displayed emission that was highly dependent on the number of layers deposited. In the **Tb**·**13** film, a total of 6 layers were deposited but maximum luminescence was observed at 4 layers. Similarly for **Tb**·**14** a total of 4 layers were deposited, but maximum emission was observed for 2 layers. The quenching of emission on additional layer deposition was ascribed to the inner filter effect [18]. Therefore, in this system it was not the film formation that resulted in quenching, but the successive deposition of films.

**Fig. 7:**
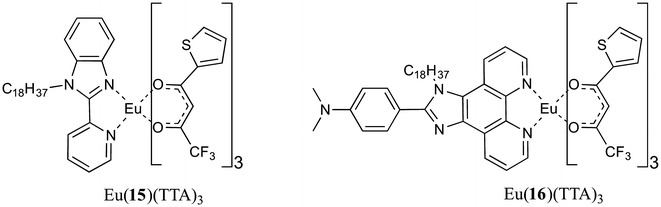
Pre-formed complexes of [Eu(TTA)_3_(**15**)] and [Eu(TTA)_3_(**16**)]

Wang and co-workers carried out an interesting study investigating the emission from films deposited at different surface pressures [19]. The pre-formed complex, [Eu(TTA)_3_(**15**)] (TTA = thenoyltrifluoroacetone, Fig. 7), formed stable Langmuir films on a pure water sub-phase. However, whilst the LB films transferred at lower pressure (12 mN m^−1^) displayed reasonable emission, the films transferred at higher pressure (30 mN m^−1^) resulted in significant quenching of emission. This observation was attributed to aggregation of luminophores within the LB film, showing that altering film formation parameters can dramatically influence the photophysical properties of the Ln(III) amphiphiles. Such aggregation induced quenching appears highly ligand dependent as the same group also reported the synthesis of the phenanthroline based complex [Eu(TTA)_3_(**16**)] (Fig. 7) [20]. In this case LB films formed at 30 mN m^−1^ gave multi-layer LB films that displayed strong emission, with no evidence of aggregation induced quenching. The examples discussed above emphasise that both ligand choice *and* film formation parameters can significantly affect the emission properties of the LB film, therefore multiple factors must be investigated/considered in ligand design.

**Fig. 8:**
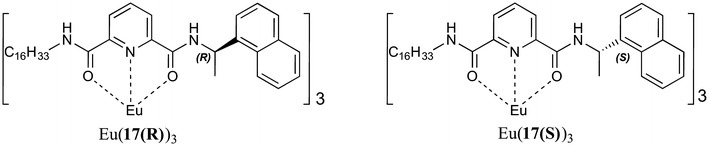
Pre-formed chiral complexes [Eu(**17(R)**)_3_] and [Eu(**17(S)**)_3_] developed by Gunnlaugsson et al

Gunnlaugsson and co-workers demonstrated the power of rational ligand design when fabricating films for specific purposes [21–23]. In this study the first examples of circularly polarised luminescence (CPL) was reported from mono-layer LB films of the chiral complexes [Eu(**17(R)**)_3_] and [Eu(**17(S)**)_3_] (Fig. 8). The ligands were pre-designed to include a terdentate coordination pocket, a chiral sensitizing antenna for the Eu(III) ions, an aliphatic chain, and in addition allow facile formation of enantiomerically pure Eu(III) complexes. Upon transfer of the chiral pre-formed complexes to a quartz substrate, it was confirmed by circularly polarised luminescence spectroscopy that the LB mono-layer films gave rise to Eu(III) centred CPL, i.e. chirality at the metal centre was maintained on deposition.

## Ln(III) Langmuir-Blodgett film sensors

Whilst many potential applications of Ln(III) based LB films have been proposed, one application that has begun to be realised is the ability of LB films to act as sensors. The previous sections have shown that LB films of amphiphilic Ln(III) containing complexes can be obtained relatively readily and such films are reasonably homogenous in coverage with deposition that does not always adversely affect photophysical output (i.e. Ln(III) luminescence). In the following section we will explore the small number of examples that are present in the literature where these types of surfaces act as sensors.

Dutton and Conte reported LB films of octafunctionalised calix[4]resorcinarenes **13** and **14** (Fig. 6) which upon exposure to solutions of TbCl_3_ (2 × 10^−4^ M) abstract Tb(III) from solution, essentially acting as ion sequestration agents which respond to their local environment. This was an extremely important result as it showed that the formation of highly ordered LB films *does not* block the sensing component to modification from external perturbation, thus making LB films ideal for sensing [24]. However, no comment on film stability upon repeated dipping was given.

**Fig. 9:**
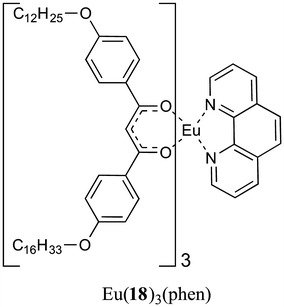
Pre-formed complex [Eu(**18**)_3_(Phen)] developed by Novikova and co-workers

In a similar type of study, Novikova and co-workers used the X-ray standing wave (XSW) technique to analyse the structural localisation of trace amounts (solutions of <10^−7^ M) of Fe, Zn, Cu and Ca ions incorporated (deliberately) into Langmuir-Blodgett films of [Eu(**18**)_3_(Phen)] (Fig. 9) on a silicon substrate [25, 26]. Whilst this study did not use emission as the output for sensing, it still reinforced the ability of LB films to respond to *very low concentrations* of analytes.

**Fig. 10:**
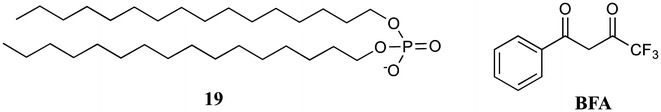
Ligand **19** was used in conjunction with Eu(III) to detect BFA

Serra and co-workers reported the ability of in situ prepared Eu(III) containing Langmuir-Blodgett films of **19** (Fig. 10) to respond to the organic compound, 4,4,4-trifluoro-1-phenyl-1,3-butanedione (BFA) [27]. When coordinated to Eu(III), this chelate is able to more effectively sensitise emission than **19** alone, therefore upon dipping the substrate coated in **19**·**Eu(III)** into an aqueous solution of BFA there was a two-fold increase in emission intensity, indicating that BFA coordinated to the Eu(III) within the LB film. This study highlighted the dynamic nature of the Eu(III) ions in LB film, as they were able to change coordination sphere and hence act as sensors to BFA. It should be noted that no comment on the stability of the LB films to dipping in the solution of BFA was given.

**Fig. 11:**
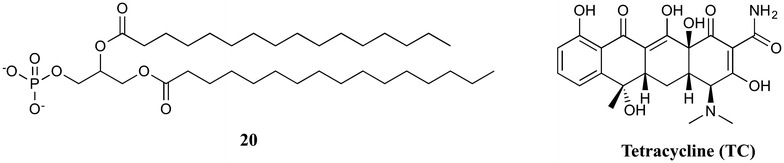
Ligand **20** used by Caminati and Puggelli to detect trace amounts of the antibiotic tetracycline (TC)

In a more application-focused example, Caminati and Puggelli utilised Eu(III) LB films for the photophysical detection of trace amounts of the antibiotic tetracycline (TC) in solution [28]. Multilayered LB films consisting of Eu(III) cations and **20** (Fig. 11) on substrates were dipped into solutions containing TC and then analysed using emission spectroscopy. No emission from Eu(III) was detected in the absence of TC, however, in the presence of TC (and with excitation at the absorption wavelength of TC) the characteristic sharp emission peaks of Eu(III) were observed. Using this technique, concentrations as low as 1 × 10^−8^ M of TC could be effectively detected. This study confirms the ability of Ln(III) amphiphiles to act as highly sensitive luminescent sensors for trace amounts of biologically relevant analytes, but the stability of the sensing films was not explicitly discussed. However, it is noted that the LB films were exposed to pH = 4 conditions with no report of degradation.

## Conclusions and future perspective

In this very brief mini-review, we have attempted to highlight the small number of LB films constructed from amphiphilic lanthanide complexes, in which at least one of the complexing ligands contains a covalently bonded amphiphilic moiety. Of the small family of Ln(III) amphiphilic systems made from both simple (e.g. **1**–**5, 19, 20**) and complex (e.g. **6**–**18**) ligands the film forming abilities have been studied in detail. This has led to an understanding of the fundamental affect/s that the lanthanide cations have on the LB films and the effect that the LB film environment has on the properties (luminescence) of the Ln(III) cations. Despite an understanding of fundamental properties, the application of these systems for advanced materials (e.g. surface bound sensors, molecular logic gates/molecular electronics) is still in its infancy. Given the retention of Ln(III) emission and good film coverage afforded by the LB method combined with initial sensing studies, the future of amphiphilic Ln(III) systems immobilised as LB films will no doubt be rich.

## Abbreviations

LB: Langmuir-Blodgett
BFA: 4,4,4-trifluoro-1-phenyl-1,3-butanedione
TTA: thenoyltrifluoroacetone
XSW: X-ray standing wave
TC: tetracycline
CPL: circularly polarised luminescence
NIR: near-infrared
